# Histopathological study of the combination of metformin and garlic juice for the attenuation of gentamicin renal toxicity in rats

**DOI:** 10.12861/jrip.2013.07

**Published:** 2013-03-01

**Authors:** Azar Baradaran, Mahmoud Rafieian-kopaei

**Affiliations:** ^1^Department of Nephrology, Division of Nephropathology, Isfahan University of Medical Sciences, Isfahan, Iran; ^2^Medical Plants Research Center, Shahrekord University of Medical Sciences, Shahrekord, Iran

**Keywords:** Gentamicin, Nephrotoxicity, Metformin, Garlic, Antioxidents, Renoprotection

## Abstract

**Introduction:** Tubular toxicity is one of the most important side effects of aminoglycoside antibiotics, especially gentamicin.Objectives: We histopathologically studied the effect of garlic extract and metformin co-administration, in attenuation of genetamicin induced tubular toxicity in rats.

**Materials and Methods:** In this study seventy rats were divided into seven equal groups and except group 1 (control) were injected 100 mg/kg/day gentamicin (GM) intraperitoneally (i.p.) for 10 days. Other than GM, group III received 20 mg/kg garlic (i.p.), group IV metformin (MF) (100 mg/kg, orally), group V a combination of MF with garlic juice (100 and 20 mg/kg/day, respectively) and group VI a combination of MF and garlic juice (50 and 10 mg/kg/day, respectively) for following 10 days. Group VII received a combination of MF and garlic juice (100 and 20 mg/kg, respectively) along with GM. Animals were sacrificed on the 20^th^ day of the experiment and the kidneys were removed for histological examinations.

** Results:** GM induced nephrotoxicity and garlic or MF alone and a combination of both with high doses (not low doses) significantly abolished the kidney tubular injury induced by GM. In addition, co-administration of GM, MF and garlic (group 7) prevented the GM- induced tissue damage more than the groups in which MF and garlic were injected 10 days post GM administration.

**Conclusion:** Garlic extract and Metformin, alone or in a combination, might be safely used to ameliorate GM induced tubular toxicity.

**Implication for health policy/practice/research/medical education:**

Garlic extract and Metformin, alone or in a combination, might be safely used to ameliorate gentamicin induced tubular toxicity.

## Introduction

Renal tubular toxicity is one of the most important adverse effects as well as therapeutical limitations of aminoglycoside antibiotics, particularly gentamicin (GM). Aminoglycoside nephrotoxicity has been found to result mainly from tubular damage (1). Therefore, the goal of reducing or protecting against aminoglycosides renal toxicity has attracted much effort and attention (1,2). Generation of oxygen free radicals is the main cause of gentamicin renal toxicity. Various free radical scavengers have also been shown to ameliorate the nephrotoxicity induced by GM (3,4). Plants have provided remedies for human maladies from ancient times (5). Herbs are often administered in combination with therapeutic drugs, which may raise the potential of herb-drug anti-oxidant activity. Indeed recent trends in controlling and treating diseases tend to favor natural antioxidant compounds rather than synthetic ones (5). Garlic is a commonly worldwide used food and its medical properties have been well recognized for centuries (6). Garlic is known for its properties, as an antioxidant against free radicals (6,7). There are several reports indicating that metformin (MF) is capable of preventing oxidative stress-induced death in cell types through a mechanism dependent on the mitochondrial permeability (8-11). Thus, metformin has the potential of protecting gentamicin-induced tubular injury (10-16). Antioxidants are usually safe and effective agents. Therefore, they seem to be good candidates for testing in human. In addition, the use of medicinal plants along with synthetic drugs is very common, and may potentiate their antioxidant properties. However, their combination effects need to be tested.

## Objectives

In this study the effect of garlic extract and metformin co-administration was tested to evaluate their ameliorative effects on tubular toxicity indeced by genetamycin in Wistar rats.

## Materials and Methods

### Drugs and chemical

Metformin (Hexal; Germany) was prepared and disolved in distilled water to be given as a single daily oral dose (100 mg/kg/day) (17). The protocol employed for gentamicin therapy has been previously reported (17).

### Plant extraction

Fresh garlic was prepared from a local grower in Hamadan (Iran), in May 2011. The garlics were chopped, crushed and for extraction they were macerated with ethanol (96%) for 48 h. Then, it was centrifugated at 200 g for 5 minutes to remove the debrises. The supernatant was filtered and evaporated at 40^o^C using a Rotary evaporator. The extract was stored at -20^o^C. The frozen extract was then reconsracted with saline to prepare final concentration when needed (7).

### Determination of total flavonoids

Total flavonoids in the extract were evaluated using the method of Sharafati-chaleshtori and coworkers (18) with minor modification. In this method, half mL of the extract or rutin (as standard flavonoid compound) was added to 1.5 mL of methanol, 0.1 mL of 1 *M* potassium acetate, 0.1 mL of 10% aluminum chloride, and 2.8 mL of distilled water and left at room temperature for 30 minutes. The reaction absorbance of the mixture was evaluated at 415 nm using rutin solutions at concentrations of 25–500 ppm in methanol. The experiment was repeated three times. The amount of total flavonoids was expressed in terms of rutin equivalents (in mg/g).

### Mesearment of total phenolic components

Total phenolic components in the extract was evaluated using the Folin–Ciocalteu method described by Bahmani and coworkers (2012) (19), with minor modification. In brief, half mL of the extract or gallic acid (as standard phenolic compounds) was added to 4 mL aqueous Na_2_CO_3_ (1 *M*) and 0.5 mL Folin–Ciocalteu reagent (1:10 diluted with distilled water). The mixture then was left for 15 minutes, and the total phenoli compounds were determined by colorimetry at 765 nm. A standard curve was prepared using 0 to 250 mg/L solutions of gallic acid in methanol:water (50:50, vol/vol). The experiment was repeated for three times and the total phenolic compounds was expressed in terms of gallic acid equivalent ( in mg/g).

### Mesearment of antioxidant capacity in the extract

The method of ferric thiocyanate was used to determined the antioxidant capacity of the extract (20). Five hundred µg of garlic extract was dissolved in ethanol, in a suitable vial, and added to a reaction mixture containing 9 mL of 40 m *M* phosphate buffer and 2.9 mL of 2.5% linoleic acid. Then, the vial content was incubated at 40^o^C for 96 hours. During incubation period every 12 hours, 0.1 mL of the vial content was diluted with 0.1 mL of ammonium thiocyanate, 9.7 mL of 75% ethanol and 0.1 mL of FeCl_2_. The sample absorbance was then measured at 500 nm and the percentage inhibition (the inhibit capacity of peroxide formation in linoleic acid) was evaluated employing the following equation;

Percentage of inhibition = [1-(absorbance of sample/absorbance of control]) ×100

A high level of inhibition percentage indicates a high antioxidant activity. Ethanol within the sample and without reagents was used as the negative control.

### Allicin measerment in the extract

The allicin content of the extract was determined using the method of Miron *et al*. (21). In brief, 200 mg of the extract and 1.0 m of 2-nitro-5-thiobenzoate (1.2 ×10^-4^*M* ) were added to 50 mM sodium phosphate and 1 mM EDTA (pH 7.2) (0.1 mL). The decrease in optical density at 412 nm was evaluated after 30 minutes incubation at room temperature (Shirzad and coworkers (18) . The allicin concentration was determined according to the following equation:

$$C_{allicin}(mg/ml)=\frac{\Delta A_{412}\times 162}{28.300}=\Delta A_{412}\times 5.7\times 10^{-3}$$

In the above formula, ΔA412 is the decrease in optical density in comparison to the initial absorption at 412 nm.

### Animals

Seventy male Wistar rats (200-250 g) were purchased from Ahvaz Jundishapur University of Medical Sciences, Ahvaz, Iran. The animals had free access to food and water (standard pelleted diet and tap water), were housed at a controlled temperature (25±3°C) and humidity (50-60%) environment with a 12 hours dark-light cycle (lights on at 7 AM). During the experiment, their general health and activities were monitored closely.

### Experimental Design

In this study 70 rats were designated into seven equal groups and were injected saline (control) or 100 mg/kg/day gentamicin (GM) intraperitoneally (i.p.) for 10 days. Other than GM, group III received 20 mg/kg garlic (i.p.), group IV metformin (MF) (100 mg/kg, orally), group V a combination of MF with garlic juice (100 and 20 mg/kg/day, respectively) and group VI a combination of MF and garlic juice (50 and 10 mg/kg/day, respectively) for following 10 days. Group VII received a combination of MF and garlic juice (100 and 20 mg/kg, respectively) along with GM. Animals were sacrificed on the 20^th^ day of the experiment the rats were sacrificed under general anesthesia by injection of ketamine (i.p.) and the kidneys were removed for histological examinations.

### Histopathological examinations

The kidneys of each animal were dissected out and fixed in buffered formalin for 12 hours and processed for histopathological examination. Three-μm-thick paraffin sections were stained with hematoxylin and eosin (H and E) for light microscope examination using conventional protocol. Histopathological studies were performed under a light microscope. Slides were coded and examined by a histopathologist who was blinded to the treatment groups. All specimens were examined for six morphological parameters including epithelial cell vacuolization, degeneration, tubular cell flattening, hyaline cast, tubular dilatation, and debris materials in tubular lumen on a semi-quantitative score from 1 to 5, while the score of zero was assigned to the normal tissue without damage .

### Ethical issues

The experiment was conducted in accordance with the National Institute of Health guide for the careful use of laboratory animals .The protocol was confirmed by the Ethical Committee of Sharekord University of Medical Sciences, Shahrekord, Iran.

### Statistical Analysis

The data were recorded as mean±SEM. The Kruskal-Wallis and Mann-Whitney U tests were applied to compare the pathology damage score between the groups. P<0.05 was considered as statistically significant.

## Results

### Bioactive components of garlic extract

The amount of flavonoids in garlic extract was 6.1±0.5 mg/g (equivalent to rutin) and the amount of phenolic compounds was 12.9±0.8 mg/g (equivalent to gallic acid). The amount of allicin in garlic extract was found to be 15 µg/mL and the antioxidant activity (the percentage of inhibition or the capacity to inhibit the peroxide formation in linoleic acid was 52.6%.

### The effect of metformin on damage score

**Figure 1:**
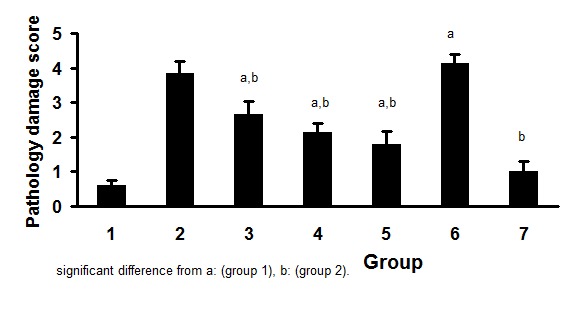
The pathology damage score in seven groups of animals.

The pathology damage score for all experimental groups are demonstrated in Figure 1. The results were compared with negative and positive control groups. The best result was obtained from the group 7 which indicated no significant difference in pathology damage score from negative control group. These data revealed that co-administration of appropriate doses of metformine and garlic extract with gentamicin vanished the gentamicin induced nephrotoxicity.

The groups 3 to 7 were compared separately with the group 1 (sham) or group 2 (positive control group). The symbol a & b‏ stand for significant difference from negative (sham) and positive controls groups (p<0.05).

Group 1, sham group; group 2, positive control group treated with gentamicin; group 3 treated with gentamicin for 10 days and post treatment with garlic extract (i.p) for next 10 days; group 4 treated with gentamicin for 10 days and post treatment with metformine (oral) for next 10 days; group 5**,** treated with gentamicin for 10 days and post treatment with combination of high doses of metformine (oral) and garlic extract (i.p) for the next 10 day; group 6, treated with gentamicin and combination of low doses of metformine (oral) and garlic extract (i.p) for 10 days, and group 7, treated with gentamicin and combination of high dose of metformine (oral) and garlic extract (i.p) for 10 days.

## Discussion

This study was aimed to determine the protective effect of garlic, metformin and their combination on kidney tissue damage induced by gentamicin. Low doses of garlic (10 mg/kg) and metformin (50 mg/kg) (co-administration) did not reduce the induced kidney tissue damage by metformin. However, MF (100 mg/kg) alone, garlic (20 mg/kg) alone, and their combination with the same dosages, significantly decreased the kidney tissue damage induced by gentamicin. In addition co-administration of mtformin and garlic and gentamicin (group 7) prevented the GM- induced tissue damage more than other the groups in which garlic and metformin were administered 10 days after gentamicin administration.

Herbs when co-administered with therapeutic drugs may potentiate the herb-drug anti-oxidant activities. In group 7, in which garlic and metformin were co-administered, the results were better than group 5 in which higher doses of garlic and metformin were administered following induction of renal toxicity.

Gentamicin is still widely used against gram-negative aerobic bacterial infections. However, due to renal impairment, which may occur in up to 30% of treated patients, its use has become limited (1,2). Moreover, gentamicin is employed as a model to study the acute renal failure in experimental animals (1,2). Gentamicin is usually accumulated in epithelial tubular cells and cause a range of effects starting with loss of the brush border in epithelial cells and activation of apoptosis, ending in overt tubular necrosis and massive proteolysis (1-4). It may also cause cell death by extracellular calcium-sensing receptor stimulation, phospholipidosis, generation of free radicals, energetic catastrophe, inflammation and reduced renal blood flow (2-4).

Various antioxidents and drugs have been shown to reduce gentamicin induced nephrotoxicity. Because of their relative effectiveness and safety, antioxidants seem to be good candidates for testing in humans. To the best of our knowledge, this is the first study in which the combination of garlic and metformin were applied for the treatment of gentamicin tubular toxicity.

The results of this study showed that garlic possesses antioxidant properties against free radicals.The protective effect of the garlic-derived antioxidant S-allylcysteine on renal injury and oxidative stress induced by ischemia and reperfusion was shown by Segoviano-Murillo *et al*. (22). In another study, Pedraza-Chaverrí and coworkers suggested that S-allylmercaptocysteine (one of the water soluble organo-sulfur compounds found in aged garlic extract) scavenges hydroxyl radical in vitro and attenuates gentamicin-induced oxidative and nitrosative stress and renal damage in vivo (23). Metformin is usually used for the treatment of diabetes (23,24). It exerts its metabolic activity through the induction of the adenosine monophosphate activated protein kinase (AMPK) pathway which acts as a sensor detecting variations of intracellular energy levels (24). Alterations in epithelial cell polarity and in the subcellular distributions of epithelial ion transport proteins are key molecular consequences of acute kidney injury and intracellular energy depletion (25). AMPK, a cellular energy sensor, is rapidly activated in response to renal ischemia, and AMPK activity may influence the maintenance or recovery of epithelial cell organization in mammalian renal epithelial cells subjected to energy depletion (25,26). At a molecular level, energy deprivation causes key energy-dependent membrane proteins to become displaced and dysfunctional (25-27). In the proximal tubule, the Na,K,ATPase is internalized from the basolateral membrane, disrupting the cell’s capacity to maintain normal transepithelial sodium transport (13,14,25-28). Inhibition of a polarized plasma membrane distribution of Na,K,ATPase in renal epithelia is essential for the maintenance of both solute reabsorption and volume homeostasis. It has been deminstrated that Na,K,ATPase becomes mislocalized after energy deprivation (25-28). ATP depletion also perturbs the distribution of tight junction proteins, further disrupting epithelial cell polarity and organization (27-29). It may lead to back leak of extracellular fluid into the urinary space. Such molecular insults result in accumulation of potentially harmful toxins (6,7). Metformin activates AMPK in rat kidney lysates (29-31). Metformin increases detectable *p*-AMPK in a dose-dependent manner, and metformin-induced AMPK activation occurs in proximal tubules as well as in distal segments (29-31). Mitochondria represents one of the major cellular sources of ROS generation (30-32) and mitochondrial toxicity may be mediated by ROS. ROS are normally produced at low levels by mitochondria themselves. However, under pathological conditions, the intramitochondrial and intracellular ROS content may be amplified (30-35). In accordance with our results, inhibition of histologic changes due to gentamicin toxicity by metformin has been shown by others (28). Control and MF-treated rats have shown no structural alterations in renal tissues, while massive and diffuse cell necrosis was observed in the proximal tubules of kidneys from rats injected with gentamicin (28). Similar results were obtained in our study.

## Conclusion

The results of our study showed that, garlic extract could be safely use together with metformin to increase the antioxident potency to ameliorate gentamicin tubular toxicity.

## Acknowledgments

The authors would like to thank all staffs of the Medical Plants Research Center, Shahrekord University of Medical Sciences for their help.

## Authors’ contributions

MRK designed and performed the research. AB prepared the final draft.

## Conflict of interests

The author declared no competing interests.

## Ethical considerations

Ethical issues (including plagiarism, data fabrication, double publication) have been completely observed by the author.

## Funding/Support

This study was granted by the research deputy of Shahrekord University of Medical Sciences (grant # 994).
